# Datasheet showing the impact of work environment on productivity in higher education institutions

**DOI:** 10.1016/j.dib.2019.104090

**Published:** 2019-06-05

**Authors:** Ifetayo Oluwafemi, Jesusetemi Oluwafemi, Timothy O. Laseinde, Bosede O. Awoyemi, Afolabi Babatunde

**Affiliations:** a Postgraduate School of Engineering Management, University of Johannesburg, RSA; b Dept. of Quality and Operations Management, University of Johannesburg, RSA; c Department of Mechanical Engineering Science, University of Johannesburg, RSA; d Department of Economics, Afe Babalola University Ado Ekiti, Ekiti State Nigeria

**Keywords:** Productivity, Work environment, Nigerian universities, Government, Management

## Abstract

This research paper provides datasheet on the summary of the investigation conducted to determine the effect of both internal and external environment on staff and students’ productivity in some selected Higher Education Institutions (HEIs) in Nigeria. It is generally acknowledged that the productivity of Nigerian HEIs is rather low, this survey examines the effect of the external environment on staff and student productivity in Nigerian HEIs, evaluates the effect of the internal environment on staff and student productivity in Nigerian HEIs, and determines the effect of psychosocial environment on staff and student productivity in Nigerian HEIs. Data were gathered based on conclusive research design. Stratified and convenience sampling techniques were adopted. The research instrument was confirmed to have all the necessary psychometric values considered appropriate for the research. Some descriptive statistical analyses were carried out to further clarify the data and provide the necessary platform for further analyses.

**Table:** Specifications table

Subject area	Business, Education, Management
More specific subject area	Total Quality Management and Human Resource Management
Type of data	Tables and Figures
How data was acquired	Field survey technics was adopted for the data collection
Data format	Raw, analyzed, Descriptive and Inferential statistical data
Experimental factors	Purposive and convenience sampling techniques were adopted
Experimental features	Work Environment is a major factor endangering Productivity particularly in Higher Education Institutions
Data source location	Southwest and North Central, Nigeria.
Data accessibility	The data is with this article
Related Research Article	Ogunnaike, O. O., Ayeni, B., Olorunyomi, B., Olokundun, M., Ayoade, O., & Borishade, T. (2018). Data set on interactive service quality in higher education marketing. *Data in brief*, *19*, 1403–1409 [1].

**Table:** 

**Value of the data** • Data can be used to examine the extent by which External Environment of Nigerian Universities Fostered Staff and Student Productivity • Data can be used to ascertain the degree of Influence the Internal Environment has on Staff and Student Productivity • Data can be used to examine if there is a significant relationship between the external environment and productivity in the Nigerian universities • Data can be used to examine the effect of Internal Work Environment on Staff and Students' Productivity in Nigerian Universities • The details of the data can be used to assess the level of learning that took place in those universities • The data provided gives an insight on the impacts work environment have on productivity of both students and staff within the confines of a corporate social responsibility in higher education institutions in Nigeria. Further studies can review this stance in another context

## 1 Data

**Fig. 1:**
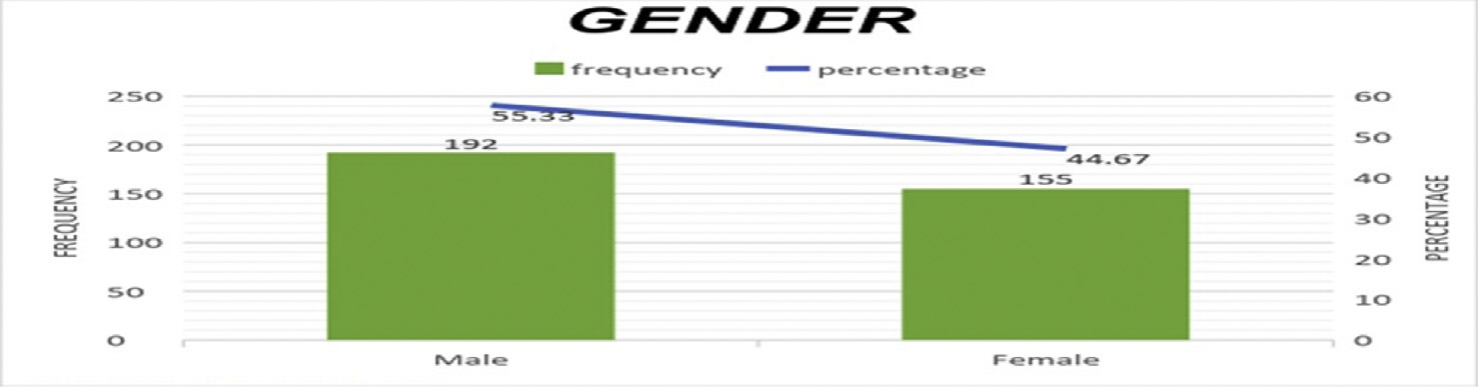
Gender distribution from seleced universities, Source:Field work 2018.

**Fig. 2:**
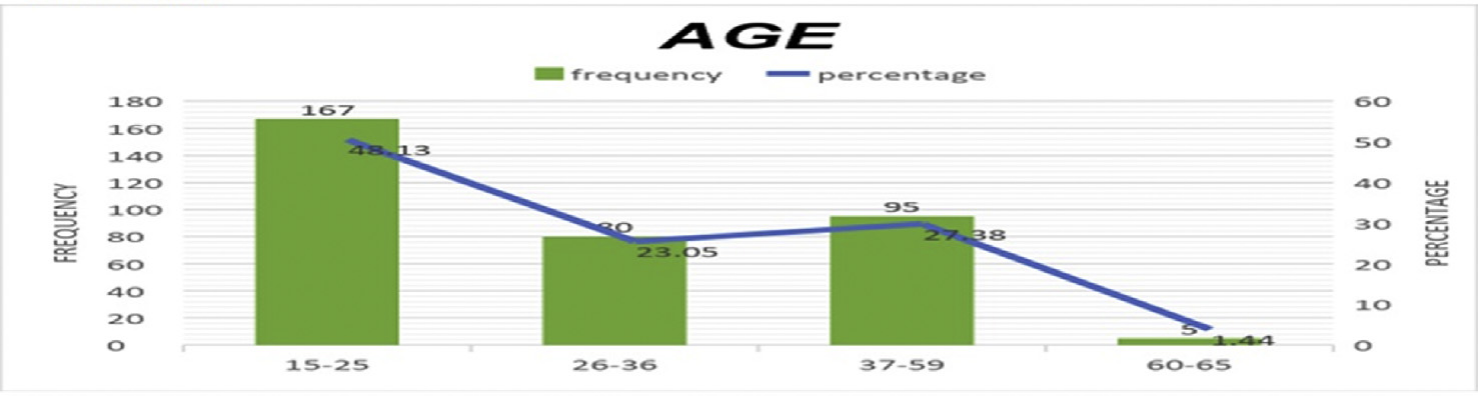
Representation of Distribution by age, Source:Field work 2018.

**Fig. 3:**
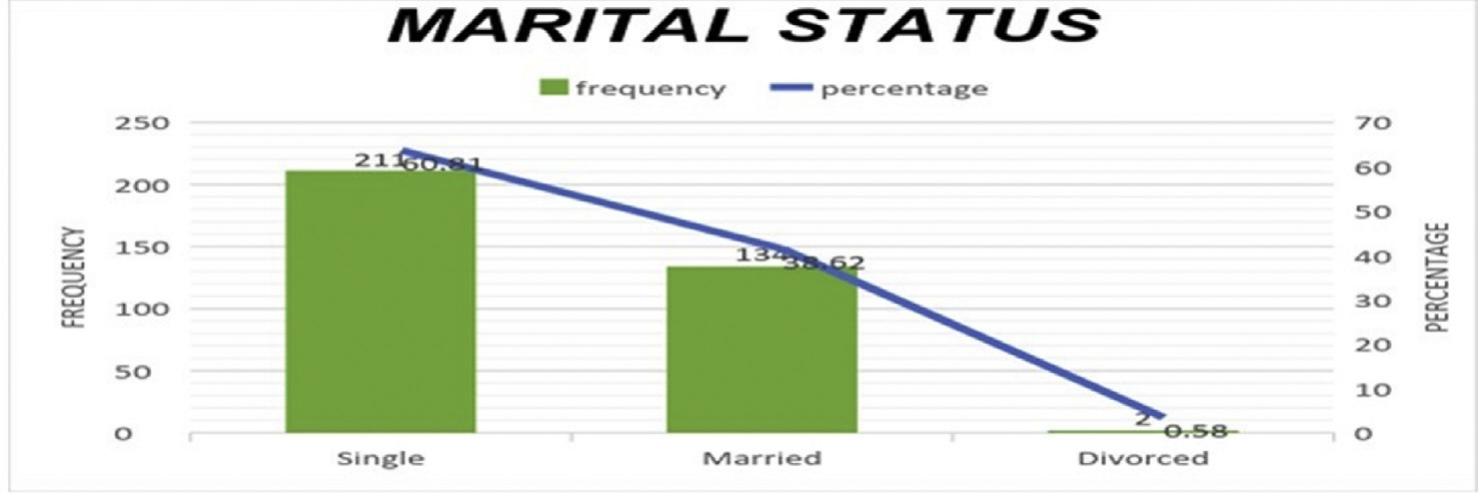
Distribution by matrial status from selected universities, Source:Field work 2018.

**Fig. 4:**
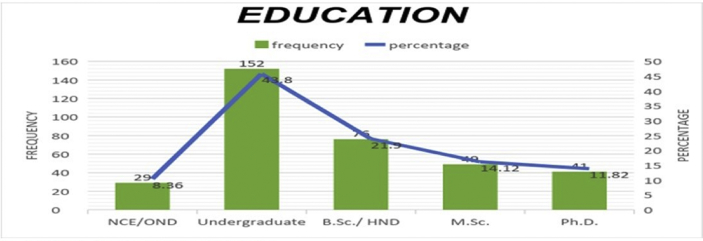
Distribution by educational qualification from selected universities, Source:Field work 2018.

**Fig. 5:**
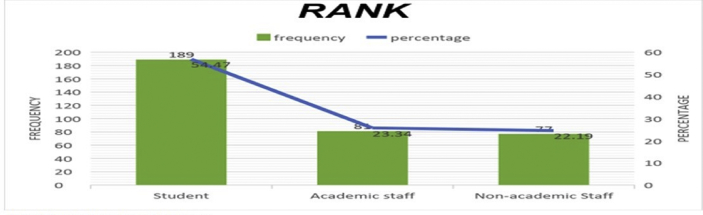
Distribution by rank from selected universities, Source:Field work 2018.

**Fig. 6:**
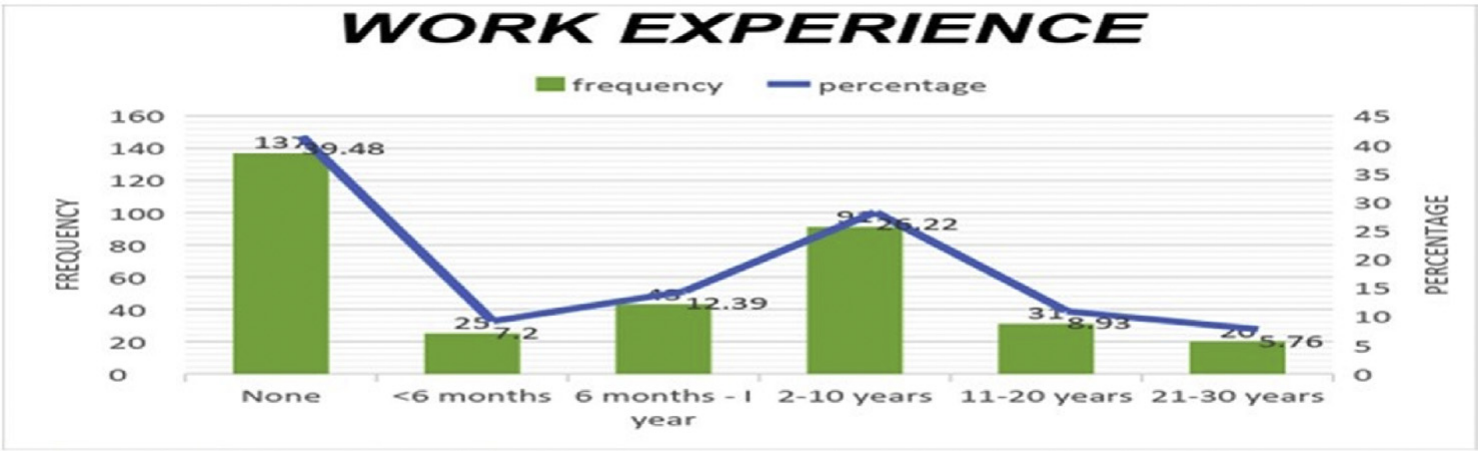
Distribution by work experience from selected universities, Source:Field work 2018.

**Fig. 7:**
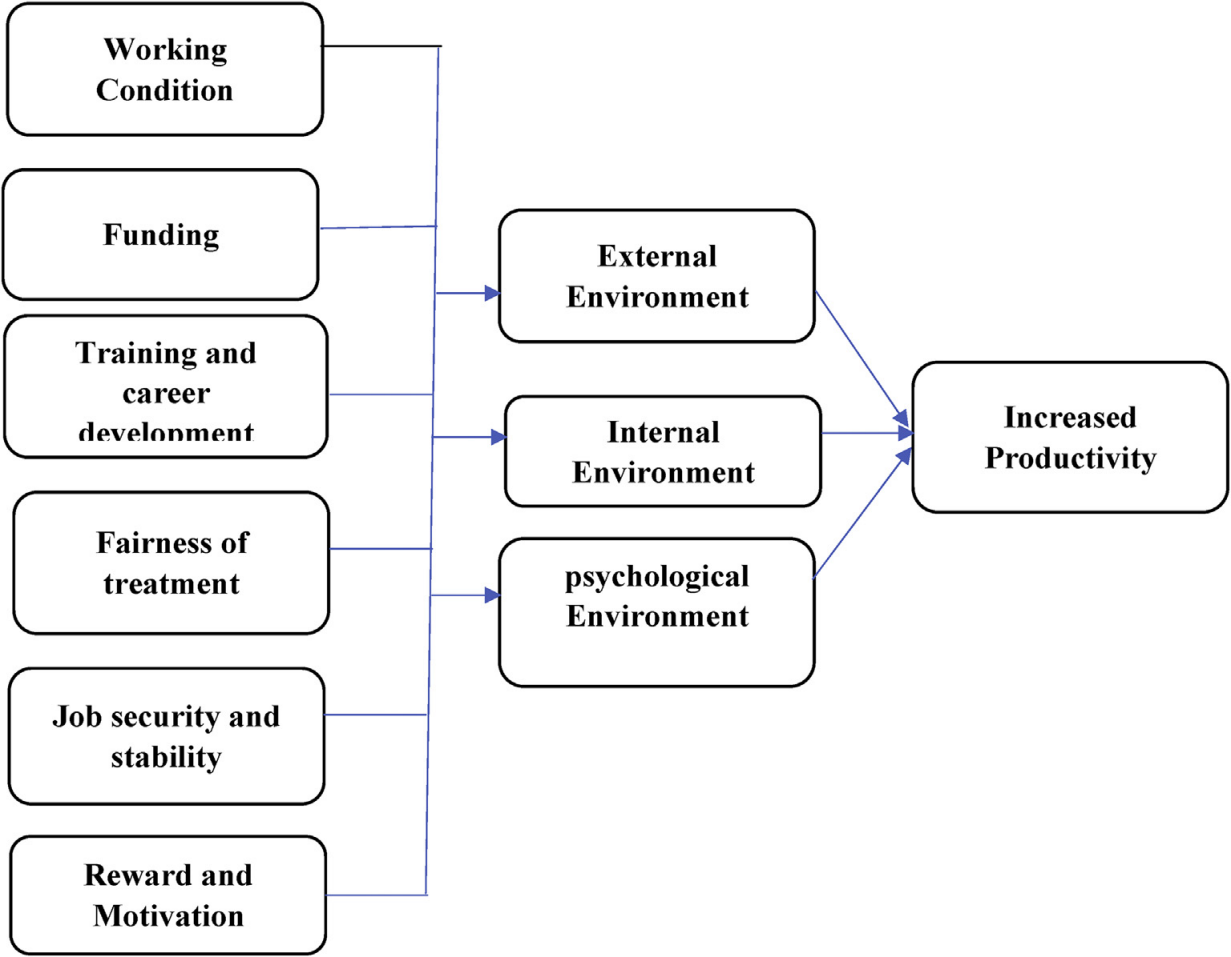
Research theoretical model.

The data presented below was obtained using a structured questionnaire. The distribution of the demographical characteristics of the respondents are presented in the bar charts below Figs. 1–6. The respondents involved in the survey were 192 male (55.33%) and 155 females (44.67%) as shown in Fig. 1. This reflects the gender distribution of the Nigerian labor force and students acquiring higher education, in which the males are larger in proportion. Fig. 2 shows 167 (48.13%) respondents were of the age 15–25, 80 (23.05%) were of age 26–36, while 95 (27.38%) fell within the range 37–59 and only 5 (1.44%) were of the range 60–65. Age 15–25 were mostly students, while some were interns working as staff, youth corps or newly employed staff. Ages 26–36 and 37–59 comprised mostly of staff with few students. Fig. 3 shows the frequency of single, married and divorced respondents, which were 211(60.81%), 134 (38.62%) and 2 (0.58%) respectively. Fig. 4 shows the academical qualification of the respondents, 29 (8.36%) had NCE/OND, 152 (43.8%) were Undergraduates, 76 (21.9%) had their B.Sc./HND, 49 (14.12%) had their M.Sc., and 41 (11.82%) had their Ph.D. Fig. 5 shows the ranks of the respondents, Fig. 6 shows the working experience of the respondents involved in the survey, 137 (39.48%) had no working experience in the educational sector; 25 (7.2%) had less than 6 months of working experience; 43 (12.39%) had worked 6 months to a year; 91 (26.22%) had worked 2–10 years; 31 (8.93%) had 11–20 years of experience; and 20 (5.76%) had 21–30 years of working experience in the sector. The theoretical model for this research is shown in Fig. 7.Fig. 7.

## 2 Research design

The research adopted a descriptive survey design in appraising the impact of work environment on the productivity of staff and students in Nigerian universities. The descriptive survey design approach was useful in surveying how work environment affects productivity of staff and students in the sampling area. Descriptive survey design method is an efficient approach of collecting data regarding characteristic of sample of a population, current practices, conditions or needs [2].

### 2.1 Target population

Target Population refers to the entire group of people, events, or things of interest that the researcher wishes to investigate. The research targeted staff and students of the six (6) selected universities in Nigeria as One hundred and thirteen thousand, three hundred and fifty-five (113, 355), as shown in Table 1. To determine the State or Federal universities to be used for analysis, 3 schools were drawn at random from a box containing a list of the top one hundred (100) NUC approved universities in Nigeria, while for private universities, this paper examined different top-ranked faith-based universities (Christian, Islam and secular) by the NUC (see Table 2, Table 3, Table 4).

**Table 1:** Population of the survey.

S/N	Schools	Type	No. Of Staff and Students
1	Afe Babalola University, Ado-Ekiti, Ekiti	Private university	8255
2	College of Education, Ikere Ekiti, Ekiti State	State University	9345
3	Al-Hikmah University, Ilorin	Private university	7499
4	Covenant University, Ota, Ogun State	Private university	16, 022
5	Ekiti State University, Ado-Ekiti	State University	45,999
6	University of Ilorin, Ilorin, Nigeria	Federal University	26,235
	**TOTAL**		**113,355**

Source: Schools Website, 2018.

**Table 2:** Sample size for each university.

S/N	Schools	Type	Total population	Sample size
1	Afe Babalola University, Ado-Ekiti, Ekiti	Private	N_i_ = 8255	n_i_ = 29
2	College of Education, Ikere Ekiti, Ekiti State	State	N_i_ = 9345	n_i_ = 32
3	University of Ilorin, Ilorin, Kwara State	Private	N_i_ = 7499	n_i_ = 26
4	Covenant University, Ota, Ogun State	Private	N_i_ = 16, 022	n_i_ = 56
5	Ekiti State University, Ado-Ekiti	State	N_i_ = 45,999	n_i_ = 160
6	University of Ilorin, Ilorin, Nigeria	Federal	N_i_ = 26,235	n_i_ = 92
	**TOTAL**		**N**_**i**_**= 113,355**	**n**_**i**_**= 398**

Source: Field Survey, 2018.

**Table 3:** Rated opinion (%) for Hypotheses One.

S/N	Statements	SA (%)	A (%)	SD (%)	D (%)	U (%)
1	My institution is committed to ensuring health and physical well-being of members	79 (22.8)	193 (55.6)	30 (8.6)	33 (9.5)	12 (3.5)
2	My institution has set structures to discourage a dirty, noisy and crowded environment	74 (21.3)	174 (50.1)	37 (10.7)	53 (15.3)	9 (2.6)
3	There are adequate equipment and facilities that encourage learning and education	65 (18.7)	157 (45.2)	39 (11.2)	79 (22.8)	7 (2.0)
4	The system provides adequate motivation to achieving set goals	69 (19.9)	180 (51.9)	34 (9.8)	51 (14.7)	13 (3.7)
5	Inadequate funding has had some negative effect on the quality of teaching and learning	164 (47.3)	122 (35.2)	27 (7.8)	29 (8.4)	5 (1.4)
6	Government funding has been grossly inadequate	142 (40.9)	130 (37.5)	20 (5.8)	25 (7.2)	30 (8.6)

Source: Fieldwork, 2018.

**Table 4:** Rated opinion (%) for Hypotheses Two.

S/N	Statements	SA (%)	A (%)	SD (%)	D (%)	U (%)
1	Plagiarism in academic is described as a serious offence	185 (53.3)	119 (34.3)	21 (6.1)	7 (2.0)	15 (4.3)
2	My institution has provisions to support staff and student development which is known to all members	57 (16.4)	147 (42.4)	69 (19.9)	47 (13.5)	27 (7.8)
3	The facilities are functional and accessible to all	41 (11.8)	135 (38.9)	72 (20.7)	73 (21.0)	26 (7.5)
4	There is adequate training on the use of these facilities	28 (8.1)	124 (35.7)	80 (23.1)	90 (25.9)	25 (7.2)
5	Workload in the system is adequately distributed	38 (11.0)	145 (41.8)	58 (16.7)	74 (21.3)	32 (9.2)
6	Clear path for career development is made known to everyone	38 (11.0)	158 (45.5)	61 (17.6)	67 (19.3)	23 (6.6)

Source: Fieldwork, 2018.

### 2.2 Sampling and sampling technique

Random sampling technique was used to carefully observe the population and ensure that everyone was well represented. Taro Yamane (1967) statistical formula was applied in extracting the sample size from the population of One hundred and thirteen thousand, three hundred and fifty-five (113, 355) respondents.

## 3 Data collection instruments and procedure

The researcher adopted the use of questionnaires in collecting data for this survey. The researcher used a drop and pick later system in the administering the questionnaire. A pilot survey was conducted to ensure the questionnaire yield consistent results. This involves a pretesting survey of staff and students of Federal Polytechnic Ado-Ekiti, Ekiti, Nigeria, which is not included in the research sample in order to ensure enough precision. This ensured that the measure actually measures what is claimed. Also, the respondent was provided with consent form to sign before completing the questionnaire, which assured them that their responses will be held in the strictest confidence. Signing and submission of the consent form constitutes implied consent to take part in the survey and to use the data provided.

### 3.1 Data analysis technique

Information that was collected through the questionnaires was thoroughly examined and streamlined because of some omission errors in answering some of the questions. Data analysis entails using categorization, tabulation, examination; these tools help in representing data information that will be gathered.

The procedures adopted for the analysis of the hypothesis used in this work are simple percentages and chi-square (χ^2^). The simple percentages were used in determining the number of respondents who either strongly agreed, agreed, strongly disagreed, disagreed or were undecided for each question, and this was presented in a tabular form. It was also used to determine the number of respondents that fell into each category (i.e., gender, marital status, age, education, rank and work experience), of which was presented using histogram. Chi-Square (χ^2^) analysis was carried out with the aid of statistical package for social sciences (SPSS) to analyze the data collected from questionnaires while Microsoft Excel was used to analyze the demography.

### 3.2 Research questions

**To What Extent Has the External Environment of Nigerian Universities Fostered Staff and Student Productivity?**

**What Degree of Influence the Internal Environment has on Staff and Student Productivity?**

### 3.3 Validity and reliability test

To ensure that the questionnaire captures what it is assumed to measure, the content validity method was used, and this method enables the questionnaire to be reviewed by professionals before its distribution it to the respondents. After certifying the correctness of the instrument, the reliability test was conducted using Cronbach's alpha. This test was conducted in order to ensure the internal reliability of the measurement. As presented in Table 5, all the variables are reliable since their Cronbach's alpha is greater than 0.60 as recommended by Al-alak and Tarabieh [3] (see Table 6, Table 7, Table 8, Table 9, Table 10, Table 11, Table 12, Table 13, Table 14).

**Table 5:** Validity and reliability test.

S/N	Variables	Cronbach's Alpha	No of Item
1	To what extent has the external environment of Nigerian universities fostered staff and student productivity?	0.853	6
2	What degree of influence the internal environment has on staff and student productivity?	0.922	6
3	External work environment on staff and students' productivity in Nigerian universities	0.781	6
4	Effect of internal work environment on staff and students' productivity in Nigerian universities	0.935	6
5	Effect of psychosocial work environment on staff and students' productivity in Nigerian universities	0.845	6

Source: Fieldwork, 2018.

**Table 6:** Observed frequency table.

Objectives	SA	A	SD	D	U	Row total
My institution is committed to ensuring health and physical well-being of members	79	193	30	33	12	**347**
My institution has set structures to discourage a dirty, noisy and crowded environment	74	174	37	53	9	**347**
There are adequate equipment and facilities that encourage learning and education	65	157	39	79	7	**347**
The system provides adequate motivation to achieving set goals	69	180	34	51	13	**347**
Inadequate funding has had some negative effect on the quality of teaching and learning	164	122	27	29	5	**347**
Government funding has been grossly inadequate	142	130	20	25	30	**347**

Source: Fieldwork, 2018.

**Table 7:** Expected Count table.

Objectives	SA	A	SD	D	U
1	101.1	144.9	39.3	47.6	14.1
2	101.1	144.9	39.3	47.6	14.1
3	101.1	144.9	39.3	47.6	14.1
4	101.1	144.9	39.3	47.6	14.1
5	101.1	144.9	39.3	47.6	14.1
6	101.1	144.9	39.3	47.6	14.1

Source: Fieldwork, 2018.

**Table 8:** χ2 calculated for External environment and staff and students’ productivity in Nigerian Universities.

Tested items	No of observations	χ2-calculated	Degree of freedom (r-1) (c-1)	Critical value	Asymptotic significance (2-sided)	χ2-Tabulated	Remark
**External environment and productivity**	4164	8.807E2^a^	44	**0.05**	**0.000**	1.960	**Reject H**_**O1**_

Source: Fieldwork, 2018.

**Table 9:** Observed frequency table. Source: Fieldwork, 2018.

Objectives	SA	A	SD	D	U	Row total
Plagiarism in academic is described as a serious offence	15	119	21	7	15	**347**
My institution has provisions to support staff and student development which is known to all members	57	147	69	47	27	**347**
The facilities are functional and accessible to all	41	135	72	73	26	**347**
There is adequate training on the use of these facilities	28	124	80	90	25	**347**
Workload in the system is adequately distributed	38	145	58	74	32	**347**
Clear path for career development is made known to everyone	38	158	61	67	23	**347**

**Table 10:** Expected Count table.

Objectives	SA	A	SD	D	U
1	55.2	151.6	55.0	52.1	33.1
2	55.2	151.6	55.0	52.1	33.1
3	55.2	151.6	55.0	52.1	33.1
4	55.2	151.6	55.0	52.1	33.1
5	55.2	151.6	55.0	52.1	33.1
6	55.2	151.6	55.0	52.1	33.1

Source: Fieldwork, 2018.

**Table 11:** χ2 calculated for Internal environment and staff and students’ productivity in Nigerian Universities.

Tested items	No of observations	χ2-calculated	Degree of freedom (r-1) (c-1)	Critical value	Asymptotic significance (2-sided)	χ2-Tabulated	Remark
Internal environment and productivity	4164	5.962E2^a^	44	**0.05**	**0.000**	1.960	Reject H_O2_

Source: Fieldwork, 2018.

**Table 12:** Observed frequency table.

Objectives	SA	A	SD	D	U	Row total
Good relationship among colleagues' aids performance	190	133	13	5	6	**347**
There must be controlled relationship between staff and students	128	188	18	2	11	**347**
Controlled interpersonal relationship amongst staff and students improve learning and education	129	174	24	5	15	**347**
Social interaction between male and female members should be controlled	79	162	53	32	21	**347**
Establishment of quality assurance team improves staff and student's performance	108	182	27	12	18	**347**
Cash rewards motivate productivity	127	144	40	14	22	**347**

Source: Fieldwork, 2018.

**Table 13:** Expected Count table.

Objectives	SA	A	SD	D	U
1	114.2	157.4	36.2	18.2	20.8
2	114.2	157.4	36.2	18.2	20.8
3	114.2	157.4	36.2	18.2	20.8
4	114.2	157.4	36.2	18.2	20.8
5	114.2	157.4	36.2	18.2	20.8
6	114.2	157.4	36.2	18.2	20.8

Source: Fieldwork, 2018.

**Table 14:** χ2 calculated for psychosocial environment and staff and students’ productivity in Nigerian Universities.

Tested items	No of observations	χ2-calculated	Degree of freedom (r-1) (c-1)	Critical value	Asymptotic significance (2-sided)	χ2-Tabulated	Remark
Psychosocial environment and productivity	4164	4.871E2^a^	44	**0.05**	**0.000**	1.960	Reject H_O3_

Source: Fieldwork, 2018.

## 4 Hypotheses testing

The hypotheses formulated for the research was tested using Chi-Square test (χ2) statistics.

### 4.1 Hypotheses one

Ho: There is no significant relationship between the external environment and productivity in the Nigerian universities.

Hi: There is a significant relationship between the external environment and productivity in the Nigerian universities.

Cross Tabulation for Testing the Effect of External Work Environment on Staff and Students’ Productivity in Nigerian Universities.

### 2.6 Hypotheses two

Ho: There is no significant relationship between the internal environment and productivity in the Nigerian universities.

Hi: There is a significant relationship between the internal environment and productivity in the Nigerian universities.

Cross Tabulation for Testing the Effect of Internal Work Environment on Staff and Students’ Productivity in Nigerian Universities.

### 2.7 Hypotheses three

Ho: There is no significant relationship between the psychosocial environment and productivity in the Nigerian universities.

Hi: There is a significant relationship between the psychosocial environment and productivity in the Nigerian universities.

**Cross Tabulation for Testing the Effect of Psychosocial Work Environment on Staff and Students’ Productivity in Nigerian Universities**.

## References

[1] Ogunnaike O.O., Ayeni B., Olorunyomi B., Olokundun M., Ayoade O., Borishade T. (2018). Data set on interactive service quality in higher education marketing. Data in brief.

[2] Chandran Emil (2004). Research Methods: A Quantitative Approach with Illustrations from Christian Ministries.

[3] Al-alak B.A., Tarabieh S.A. (2011). Gaining competitive advantage and organizational performance through customer orientation, innovation differentiation, and market differentiation. Int. J. Econ. Manag. Sci..

