# Supplementary material for: Datasheet showing the impact of work environment on productivity in higher education institutions
Source: Data Brief. 2019 Jun 5;25:104090. doi: 10.1016/j.dib.2019.104090 (PMC6598870; doi:10.1016/j.dib.2019.104090)
Supplement: Multimedia component 1 [file mmc1.docx]

**QUESTIONNAIRE’**

Dear Respondent,

I am a postgraduate student investigating the effect of Work Environment on Productivity in Nigerian Universities. I solicit your participation in realizing this objective. All information provided will be treated with utmost confidentiality. Please answer the questions accordingly. Thank you for your cooperation.

**SECTION A**

***Demography***

1. Gender: Male ( ), Female ( )
2. Marital status: Single ( ), Married ( ), Divorced ( )
3. Age: 15-25 ( ), 26-36 ( ), 37-59 ( ), 60-65 ( )
4. Education: NCE/OND ( ), Undergraduate ( ), B. Sc. / HND ( ), M. Sc. ( ), Ph. D. ( )
5. Rank: Student ( ), Academic staff ( ), Non-academic staff ( ), Administrative staff ( )
6. Work experience in the educational sector:

None ( ), < 6 months ( ), 6 months – 1 year ( ), 2-10 years ( ), 11-20 years ( ), 21-30 years ( )

**SECTION B**

Please check (x) to express your level of agreement with these statements using the following scale:

**SA** = Strongly Agree, **A** = Agree, **SD** = Strongly Disagree, **D** = Disagree, **U** = Undecided)

***External Environment***

| S/N | Questions | SA | A | SD | D | U |
| --- | --- | --- | --- | --- | --- | --- |
| 7 | My institution is committed to ensuring health and physical well-being of members |  |  |  |  |  |
| 8 | My institution has set structures to discourage a dirty, noisy and crowded environment |  |  |  |  |  |
| 9 | There are adequate equipment and facilities that encourage learning and education |  |  |  |  |  |
| 10 | The system provides adequate motivation to achieving set goals |  |  |  |  |  |
| 11 | Inadequate funding has had some negative effect on the quality of teaching and learning |  |  |  |  |  |
| 12 | Government funding has been grossly inadequate |  |  |  |  |  |

***Internal Environment***

| S/N | Questions | SA | A | SD | D | U |
| --- | --- | --- | --- | --- | --- | --- |
| 13 | Plagiarism in academic is described as a serious offence |  |  |  |  |  |
| 14 | My institution has provisions to support staff and student development which is known to all members |  |  |  |  |  |
| 15 | The facilities are functional and accessible to all |  |  |  |  |  |
| 16 | There is adequate training on the use of these facilities |  |  |  |  |  |
| 17 | Workload in the system is adequately distributed |  |  |  |  |  |
| 18 | Clear path for career development is made known to everyone |  |  |  |  |  |

***Psychosocial Environment***

| S/N | Questions | SA | A | SD | D | U |
| --- | --- | --- | --- | --- | --- | --- |
| 19 | Good relationship among colleagues aids performance |  |  |  |  |  |
| 20 | There must be controlled relationship between staff and students |  |  |  |  |  |
| 21 | Controlled interpersonal relationship amongst staff and students improve learning and education |  |  |  |  |  |
| 22 | Social interaction between male and female members should be controlled |  |  |  |  |  |
| 23 | Establishment of quality assurance team improves staff and students performance |  |  |  |  |  |
| 24 | Cash rewards motivate productivity |  |  |  |  |  |
